# CLAIMS-BASED FRAILTY INDEX AS A PREDICTOR OF POSTACUTE SKILLED NURSING FACILITY CARE OUTCOMES

**DOI:** 10.1093/geroni/igae098.1261

**Published:** 2024-12-31

**Authors:** Sandra Shi, Gahee Oh, Chan Mi Park, Ellen McCarthy, Dae Hyun Kim

**Affiliations:** Harvard Medical School/Hebrew SeniorLife, Boston, Massachusetts, United States; Hebrew SeniorLife, Boston, Massachusetts, United States; Hebrew SeniorLife, Boston, Massachusetts, United States; Harvard Medical School, Boston, Massachusetts, United States; Hebrew SeniorLife, Boston, Massachusetts, United States

## Abstract

How claims-based frailty index (CFI) predicts post-acute skilled nursing facility (SNF) care outcomes is unknown. We used the National Health and Aging Trends Study 2011-2017 survey data linked to Medicare claims. CFI and comprehensive geriatric assessment-based frailty index (CGA-FI) were calculated at each survey and categorized into non-frail (<0.25), mild frailty (0.25-0.34), moderate frailty (0.35-0.44), and severe frailty (≥0.45). We examined the first post-acute SNF admission within 6 months after survey assessment. The primary outcome was home time in the 6 months following SNF admission defined as days not in hospital or nursing home. We estimated the effects of frailty on home time using linear regression for each frailty measure. The secondary outcome was total healthcare costs in the 6 months following SNF admission estimated using a generalized linear model with a gamma distribution and identity link. Analyses adjusted for age, sex, and income. In our sample of 643 Medicare beneficiaries (representing 2.7 Million beneficiaries nationally), increasing frailty was associated with greater home time loss and healthcare costs by both measures. The mean difference (95% CI) of home time loss for severe frailty compared with non-frailty was -39.2 [-66.8, -11.5] days by CFI and -43.2 [-60.0, -26.4] days by CGA-FI. The mean healthcare cost increase (95% CI) for severe frailty compared with non-frailty was $21,583 [$13,132, $30,033] by CFI and $11,110 [$6,236, $15,983] by CGA-FI, respectively. Pearson correlation between CFI and costs was higher than CGA-FI (0.1885 vs 0.0804). CFI up to 6 months before SNF admission predicts healthcare utilization.

